# Impact of WHO Hand Hygiene Improvement Program Implementation: A Quasi-Experimental Trial

**DOI:** 10.1155/2016/7026169

**Published:** 2016-11-23

**Authors:** Farinaz Farhoudi, Anahita Sanaei Dashti, Minoo Hoshangi Davani, Nadiyeh Ghalebi, Golnar Sajadi, Raziyeh Taghizadeh

**Affiliations:** ^1^Infection Prevention and Control Unit, Nemazee Hospital, Shiraz University of Medical Sciences, Shiraz, Iran; ^2^Professor Alborzi Clinical Microbiology Research Center, Nemazee Hospital, Shiraz University of Medical Sciences, Shiraz, Iran

## Abstract

*Objectives*. As affirmed by the World Health Organization (WHO), hand hygiene is the most powerful preventive measure against healthcare-associated infections (HCAIs) and, thus, it has become one of the five key elements of patient safety program. The aim is to assess the effect of implementation of the WHO's Multimodal Hand Hygiene Improvement Strategy among healthcare workers of a tertiary teaching hospital in a developing country.* Methods*. Hand hygiene compliance was assessed among healthcare workers, according to five defined moments for hand hygiene of the WHO, before and after implementation of the WHO's Multimodal Hand Hygiene Improvement Strategy in fourteen wards of a tertiary teaching hospital in Shiraz, Iran. We used direct observation method and documented the results in WHO hand hygiene observation forms.* Results*. There was a significant change in compliance before and after implementation of WHO's Multimodal HH Improvement Strategy (29.8% and 70.98%, resp.).* Conclusions*. Implementing WHO hand hygiene program can significantly improve hand hygiene compliance among nurses.

## 1. Introduction

Healthcare-associated infection (HCAI) is one of the most important challenges of healthcare systems due to its strong impact on patient's safety and high financial burden [1–4]. In developed countries, the prevalence of HCAI is estimated between 5.1% and 11.6% [5]. The costs of HCAIs in the United States are estimated to be about $6.8 billion per year [6]. However, most reports of HCAI prevalence from developing countries are above 10%. Consequences of HAIs include increased morbidity, mortality, and expenditure [5]. Hands of healthcare personnel are known to be the main culprit of cross transmission of pathogens in healthcare facilities, and, as stated by the WHO and Centers for Disease Control and Prevention (CDC), HH is the most effective preventive measure against HAIs. Nonetheless, HH compliance among healthcare workers is not acceptable, below 40% globally [3, 7–9]. Noncompliance reasons are surveyed in different studies. They are categorized into individual, group, and institutional levels. The main reasons are the lack of education, being a medical doctor, lack of performance feedback, working in a critical care unit, lack of available or suitable HH agents, and lack of skin care products [9–12]. Today, HH has become a major issue of patient safety [6]. In addition to being a key element in standard precautions, HH has emerged as an important component in specific site infection prevention recommendations recently [13]. In accordance with the first Global Patient Safety Challenge, the WHO published important instructions including guidelines on HH in healthcare, implementation of the WHO Multimodal HH Improvement Strategy, and HH technical reference manual [14]. The aim of these guidelines is to improve HH practices worldwide by creating a unified description for HH methods, right moments, and observation process and present multimodal strategies for improvement [14–17].

Due to the lack of HH observation surveys in developing countries and the few WHO based HH observation surveys worldwide, we implemented the WHO Multimodal HH Improvement Strategy to assess its feasibility and efficacy in a developing country.

## 2. Methods

This quasi-experimental study was conducted in Nemazee Hospital, a tertiary academic center, between June 2014 and June 2015. It is the largest hospital in the south of Iran (a developing country) and a referral center for neighboring provinces with 1000 beds in 54 wards including emergency, intensive care, surgical, internal medicine, and pediatrics subspecialties. We implemented the WHO's Multimodal HH Improvement Strategy and assessed the compliance of HH before and after the interventions.

The Multimodal HH Improvement Strategy consists of five key elements that are (1) system change to ensure access of healthcare workers to HH facilities with emphasis on availability of alcohol-based hand rub (ABHR) formulations at the point of care, (2) ongoing training and education, (3) evaluation of practices and feedback, (4) reminders at the workplace, and (5) providing a climate of safety through institution [14].

The entire project included five steps: (1) facility preparedness, (2) baseline evaluation, (3) implementation, (4) follow-up evaluation, and (5) ongoing planning and review cycle [14].

In step (1), facilities were prepared. The human and financial resources were obtained, key leadership and its deputy were identified, sources were evaluated, and the strategy for the whole program was clearly defined. Hand washing sinks were adequate (one sink for up to 6 beds) and were equipped with unmedicated soap, but paper towels were not widely available. Commercially produced ABHR dispensers were located out of each room and thus there was no access to ABHR at the point of care. Educational sessions on HH were conducted only for nursing staff occasionally and although most hospital's medical doctors were not aware of HH importance and right techniques, due to cultural drawbacks, no educational session was held for them.

Baseline evaluation of HH compliance was performed, using the WHO's direct observation method. As the direct observation method is time-consuming and also due to the lack of sufficient personnel, we randomly selected 14 wards from 54 wards. All medical departments of the hospital (emergency, internal, pediatric, intensive care, and surgical) were included in these selected wards. Observation sessions were performed by 2 general medical doctors who were working as infection prevention and control practitioners and were trained by scientific- research deputy of infection prevention and control unit. The training course consisted of 2 parts. In the first part, the WHO's training Power Point slides for observers were taught during a period of about 2 hours, followed by 2 hours for completing observation forms according to WHO's sample video clips [18]. Observers were also taught important points from WHO's HH technical reference manual. In the second part, experimental form completion took place in wards under the supervision of a senior observer.

The WHO's direct observation form is based on “My Five Moments for HH” that consists of the following: before patient contact, before aseptic procedure, after body fluid exposure risk, after patient contact, and after contact with patient surroundings as HH indications [15]. A positive or negative HH action, whether hand washing or hand rubbing, was recorded provided that it related to an indication. Opportunity is defined as the time HH should happen and it must relate to at least one HH indication. The compliance is calculated by dividing positive actions by opportunities. HH practice of healthcare workers was monitored in 30–45-minute sessions (open wards and wards with multibed rooms, resp.). As it is recommended not to observe more than two healthcare workers simultaneously, HH opportunities were recorded during care sequences and, on the other hand, there was a time shortage for each session and a limited number of healthcare workers of a ward were observed. All four defined professional categories of data were recorded with focus on nurses due to their prominent role in healthcare activities. During the direct observations, the healthcare workers were aware of being observed since they knew the infection control practitioners. Each observer conducted only two sessions daily. To save time and also gather a greater number of opportunities, HH monitoring was performed at medication time when HH opportunities had the highest density. Each period of data collection lasted 3-4 weeks.

The average number of observed opportunities was 16 per ward. There was no performance feedback during the observation periods. Collected data were anonymous and were kept confidential.

In step (3), the improvement program was implemented. Bed mounted ABHR holders were designed and provided. Local production of ABHR according to WHO ethanol based formula commenced due to financial reasons and each bed was equipped with ABHR and thus alcohol-based ABHR became available at the point of care and paper towels became more available. Visual HH color posters in different sizes were provided that showed the five moments for HH and right techniques. Posters were placed in the most visible places in wards (in front of nursing stations). Five billboards were dedicated to infection prevention and control unit and were placed in strategic zones within the hospital. Infection prevention and control points with special emphasis on HH role in prevention of HAI, promotional messages, and right techniques were displayed on boards. To better attract healthcare workers attention, many messages were colorful, cartoon-like. or comic. The messages were changed monthly.

Nursing staff had to enroll in infection prevention and control educational courses (including HH topics as the main part) twice a year. The content was based on WHO's training slides. Supervisors had an extra educational session. For the first time in our hospital, infection prevention and control educational sessions were conducted for medical students before their clinical training and for staff medical doctors annually. An infection prevention and control booklet was provided and newly employed nurses had to read it thoroughly and obtain at least 70 (out of 100) in the examination based on its content; otherwise, they had to prepare for another examination.

Teaching rounds were conducted with emphasis on intensive care units due to lower compliance rate according to prior studies [4, 10, 12, 19]. In these rounds, five moments for HH and right techniques were practiced. The program was announced to medical and nursing key leaders during separate sessions. Senior hospital manager approved the project and it became one of the hospital priorities.

In step (4), after 12 months, follow-up evaluation for assessment of program effectiveness was performed. Observation feedback was announced through hospital quality improvement sessions.

Data were analyzed using SPSS version 18 and *p* value less than 0.05 was considered statistically significant.

## 3. Results

In the present study, we assessed the healthcare personnel's HH compliance in 14 wards of the hospital using the WHO's method before and after the implementation of WHO's HH improvement program through the institution [10, 17]. According to this method, an opportunity is defined as the proper time for HH according to “My Five Moments for HH” during the care sequences [10, 13, 18]. Also, we recorded actions, both hand washing and hand rubbing, according to five indications: before patient contact, before an aseptic task, after risk of exposure to body fluid, after patient contact, and after contact with patient surroundings. The compliance is calculated by dividing the number of positive actions by the number of opportunities (not indications). Before the intervention, a total of 255 opportunities (nurses: 243; auxiliaries (orderlies): 6; medical doctors: 3; others: 3) and 76 actions for HH were recorded. After the intervention, the compliance rate improved from 29.8 to 70.98% (193 opportunities and 137 actions).

**Figure 1:**
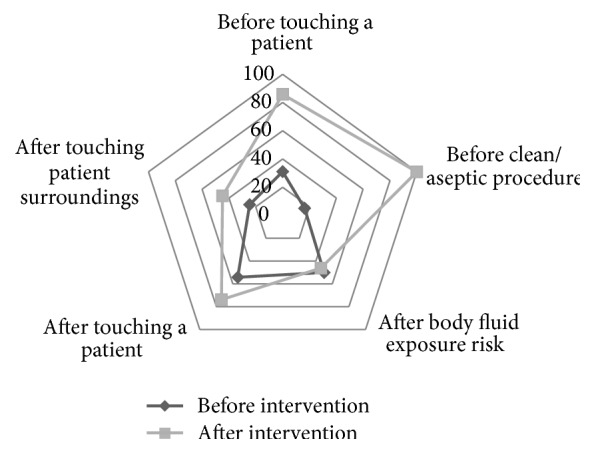
The radar chart of hand hygiene compliance by indications, before and after the intervention.

Table 1 shows that compliance with HH increased after intervention for all of the “moments of HH” except for body fluid exposure risk. Also, for ease of understanding, these differences are displayed in Figure 1.

The HH compliance rates before and after the intervention are presented in Table 2.

Due to the lack of data (small number of recorded opportunities) related to auxiliaries and medical doctors, the results of compliance ratio in nurses before and after the implementation of the program are compared.

As shown in Table 2, there is a substantial increase in observed compliance with HH practices after completing the implementation of the HH improvement program (from 29.6% to 72.7). This difference was confirmed using *χ* ^2^ test (*p* < 0.001).

**Figure 2:**
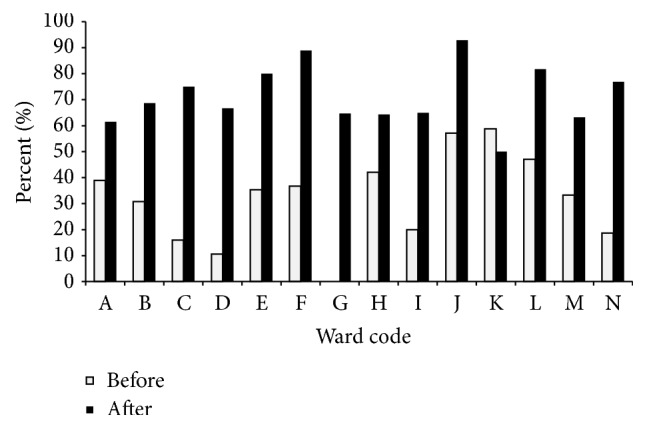
Hand hygiene compliance of nurses per ward. (A) ICU, (B) Internal Pediatrics, (C) Emergency, (D) Surgical Pediatrics, (E) Internal Pediatrics, (F) ICU, (G) Surgical, (H) Surgical, (I) Internal, (J) Internal, (K) ICU, (L) Internal, (M) Internal, and (N) ICU.

Figure 2 displays the changes in HH compliance of nurses per ward. Based on this result, the greatest difference in proportions is related to ward “G” (0% versus 64.71%) and then ward “C” (16% versus 75%) which were a surgical ward and an emergency ward, respectively.

Table 3 represents the observed opportunities and actions, both hand washing and hand rubbing, in different professional categories after the intervention. According to this table, nurses and auxiliaries contribute to HH compliance in 72.6% and 65.15% of their opportunities, respectively. Also, there are large differences between the proportions of hand rubbing and hand washing, as it seems that hand rubbing is much more popular than hand washing among the personnel (Figure 1).

**Figure 3:**
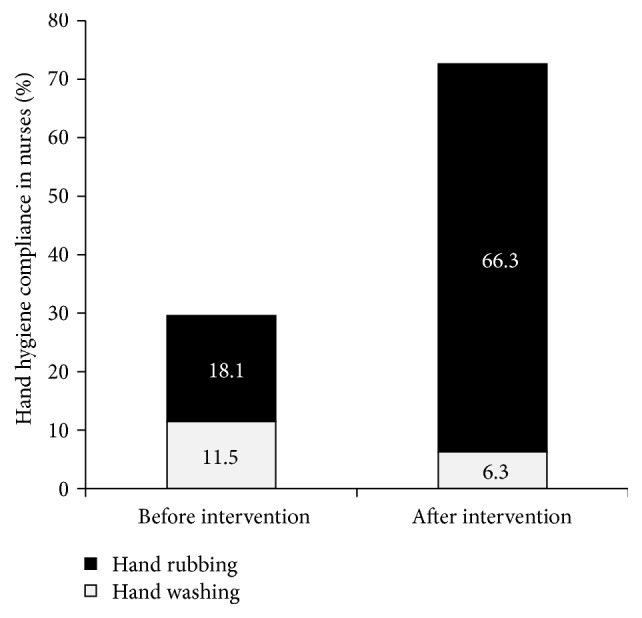
Effect of system change on the proportion of hand hygiene techniques among nurses.

Change of system by making ABHRs available at the point of care considerably improved hand rubbing proportion among nurses (*p* < 0.001) (Figure 3).

## 4. Discussion

HH practice is the single most effective measure for prevention and reduction of HCAIs [14, 16, 20].

In a recent interventional survey, healthcare-associated infection rate declined significantly and constantly from 4.8 to 3.3 (*p* < 0.01) per 1000 inpatient days, after implementation of a hospital-wide HH initiative that led to a marked improvement in staff behavior [21].

Such behavioral changes toward HH improvement need multimodal interventions including providing ABHRs and continuous educational programs as well as strong support by healthcare administrators [13]. In the present study, it was shown that implementation of WHO HH improvement program led to a significant increase in HH compliance rates in nurses of a large academic hospital.

In a quasi-experimental multicenter study, WHO strategies, including multimodal interventions, were implemented in a stepwise fashion and hand hygiene compliance of healthcare workers was assessed before and after the interventions. Furthermore, long-term sustainability of strategic activities was evaluated two years later [6]. Compliance was defined as the proportion of predefined opportunities met by hand hygiene actions (hand washing or hand rubbing). The reported compliance increased from 51.0% before the intervention (95% CI: 45.1–56.9) to 67.2% after the intervention (95% CI: 61.8–72.2). In the second assessment 2 years after the intervention, ongoing HH activities with sustained or further improvement were reported from all enrolled centers.

Some reports denote various compliance rates for the five indications of HH that are sometimes statistically different [10]. A quasi-experimental research between 2006 and 2008 at six pilot sites in Italy, Pakistan, Mali, Costa Rica, and Saudi Arabia (55 departments in 43 hospitals within these countries) stated that HH compliance with indications protecting the patient was significantly lower than indications that prevent healthcare workers from contamination and being infected; the compliance of “before patient contact” and “before clean and aseptic tasks” was the lowest and the compliance of “after exposure risk with body fluids” and “after patient contact” was the highest [6]. However, the tendency to self-protect that is reported by other studies was not evident in our study [9, 14, 22, 23].

A remarkable finding in the present study was improvement of HH compliance at all of the “moments for HH” except “after body fluid exposure risk.” The explanation could be the small number of observed opportunities of “after body fluid exposure” during the study period.

An observational survey [10] in 5 intensive care units (ICUs) and among 242 healthcare workers at a university hospital used the WHO's “five moments for HH” as the basis for observations. The overall reported noncompliance rate was 58% and the lowest HH compliance rates among opportunities were “before touching a patient” (37.3%) and “after touching a patient or the patient's surroundings” (45.3%). Among the multiple factors associated with noncompliance, the odd ratio of performance of HH “before patient contact” was the highest (OR: 4.5).

The compliance to “before clean/aseptic procedure” was poor in our center in baseline evaluation and before intervention. This finding was presented by WHO reports from other countries [3, 11, 16]. This poor condition changed dramatically after intervention and the best result in five opportunities after the intervention was observed for doing HH “before clean/aseptic procedure.” This may be due to more intense education and emphasis on it. However, this significant improvement of the mentioned indication compliance is not in concordance with other studies and shows the variable rates of compliance between indications, both before and after interventions among researches [10, 24–29].

One factor that could affect any HH improvement program is the type of ward and nature. A few investigations that have specifically assessed HH compliance in surgical wards showed the lower rate of implementation of HH in these wards [7, 29, 30], a rate that could be even 59% lower than medical wards [25]. Nevertheless, based on our results, the greatest advance in compliance after the intervention was related to a surgical ward (0% before versus 64.71% after) and then an emergency ward (16% before versus 75% after).

Another involved factor in the HH practice is the professional group. Medical doctors have generally lower HH compliance rates than nurses. Based on one study, about half of the medical doctors thought that HH is necessary after patient contact and only one-third of them believed that HH is mandatory before patient contact [31].

It is noteworthy that being a medical doctor (OR 1.712, 95% CI: 1.126–2.989) could be a significant risk factor for HH noncompliance in the hospital after adjusting for other potential risk factors [11].

In a multicenter study done in 5 countries, medical doctors had the lowest and nurses had the highest compliance before the intervention except for Costa Rica and Mali. The HH practice remained better in nurses than in medical doctors across all test sites, except for Mali [6].

Before intervention, HH compliance of medical doctors was 11.5% (52 opportunities and 6 actions) which was much lower than of the nurses (29.62%) in our study. Contrary to other studies, after intervention, HH compliance of nurses (72.6%), auxiliaries (65.15%), and medical doctors (82.35%) rose dramatically [1, 5, 6, 9, 12, 15, 32–34]. This finding of the present study could be probably due to the small number of observed opportunities among medical doctors or their better recall of previous knowledge after educational intervention.

Also, there are considerable differences between the proportions of hand rubbing and hand washing as it seems that hand rubbing is much more popular than hand washing among the personnel. This was shown in a study by Allegranzi et al. [6]. They noted a significant increase in hand rubbing method and it was the favored way of hand hygiene, after intervention, in the majority of sites. Despite the significant statistical HH improvement among nurses, this study was prone to “Hawthorne effect” due to the direct observation method and short follow-up period.

## 5. Conclusion

The fact that using WHO HH promotion strategy leads to an improvement of HH practice is shown in the present and other studies [35]. It is our belief that such interventional program in a large pilot hospital and in a developing country could serve as an acceptable model for other initiatives. The significance of HH and commitment to it should also be better taught worldwide, especially in countries with lower compliance and higher HCAIs. Hand hygiene promotion also demands more local and oriented researches. Our study revealed poor HH compliance among medical doctors before the intervention, but a significant improvement after the intervention. Further investigations with sufficient sample size are needed to clarify the reasons of noncompliance among medical doctors and assess the effectiveness of the multimodal strategy in this professional group.

## Figures and Tables

**Table 1:** Hand hygiene compliance by hand hygiene indications (events) before and after the intervention regardless of professions.

Hand hygiene indications		Before intervention	After intervention
Before touching a patient	Action, *N* (%)	13 (31)	85 (85.8)
	Indication, *N*	42	99
Before clean/aseptic procedure	Action, *N* (%)	13 (16.4)	3 (100)
	Indication, *N*	79	3
After body fluid exposure risk	Action, *N* (%)	11 (50)	6 (46.1)
	Indication, *N*	22	13
After touching a patient	Action, *N* (%)	32 (54.2)	31 (73.8)
	Indication, *N*	59	42
After touching patient surroundings	Action, *N* (%)	17 (24.6)	13 (44.8)
	Indication, *N*	69	29

**Table 2:** Comparison of hand hygiene compliance of nurses.

	Opportunities, *N*	Compliance, % (95% CI)	*χ* ^2^ test statistic	*p* value
Before intervention	243	29.6 (23.86, 35.34)	55.63	<0.001
After intervention	110	72.7 (64.37, 81.02)		

**Table 3:** Observed opportunities and actions for hand hygiene after the intervention.

Professional category	Opportunities, *N*	Hand washing, *N* (%)	Hand rubbing, *N* (%)	Compliance rate, % (95% CI)
Nurse	110	7 (6.3)	73 (66.3)	72.7 (64.37, 81.02)
Auxiliary	66	5 (7.5)	38 (57.5)	65.15 (53.65, 76.65)
Medical doctor	17	0 (0%)	14 (82.3)	82.35 (64.23, 100.47)
Total	193	12 (6.2)	125 (64.7)	71 (64.58, 77.38)
		Total actions = 137		
	Compliance rate (%) = 71%			

## Abbreviations

CDC: Centers for Disease Control and Prevention
HCAI: Healthcare-associated infection
HH: Hand hygiene
WHO: World Health Organization
ABHR: Alcohol-based hand rub.
